# Clinical Impact of Respiratory Motion Correction in Simultaneous PET/MR, Using a Joint PET/MR Predictive Motion Model

**DOI:** 10.2967/jnumed.117.191460

**Published:** 2018-09

**Authors:** Richard Manber, Kris Thielemans, Brian F. Hutton, Simon Wan, Francesco Fraioli, Anna Barnes, Sébastien Ourselin, Simon Arridge, David Atkinson

**Affiliations:** 1 Centre for Medical Imaging, Division of Medicine, University College London, London, United Kingdom; 2 Institute of Nuclear Medicine, UCL and UCL Hospitals, London, United Kingdom; 3 Centre for Medical Radiation Physics, University of Wollongong, Wollongong, New South Wales, Australia; and; 4 Centre for Medical Imaging Computing, Faculty of Engineering, University College London, London, United Kingdom

**Keywords:** motion correction, PET/MRI, lesion detection, lesion quantification

## Abstract

In PET imaging, patient motion due to respiration can lead to artifacts and blurring, in addition to quantification errors. The integration of PET imaging with MRI in PET/MRI scanners provides spatially aligned complementary clinical information and allows the use of high-contrast, high-spatial-resolution MR images to monitor and correct motion-corrupted PET data. On a patient cohort, we tested the ability of our joint PET/MRI-based predictive motion model to correct respiratory motion in PET and show it can improve lesion detectability and quantitation and reduce image artifacts. **Methods:** Using multiple tracers and multiple organ locations, we applied our motion correction method to 42 clinical PET/MRI patient datasets containing 162 PET-avid lesions. Quantitative changes were calculated using SUV changes in avid lesions. Lesion detectability changes were explored with a study in which 2 radiologists identified lesions in uncorrected and motion-corrected images and provided confidence scores. **Results:** Mean increases of 12.4% for SUV_peak_ and 17.6% for SUV_max_ after motion correction were found. In the detectability study, confidence scores for detecting avid lesions increased, with a rise in mean score from 2.67 to 3.01 (of 4) after motion correction and a rise in detection rate from 74% to 84%. Of 162 confirmed lesions, 49 showed an increase in all 3 metrics—SUV_peak_, SUV_max_, and combined reader confidence score—whereas only 2 lesions showed a decrease. We also present clinical case studies demonstrating the effect that respiratory motion correction of PET data can have on patient management, with increased numbers of detected lesions, improved lesion sharpness and localization, and reduced attenuation-based artifacts. **Conclusion:** We demonstrated significant improvements in quantification and detection of PET-avid lesions, with specific case study examples showing where motion correction has the potential to affect diagnosis or patient care.

Because of the long acquisition duration of PET (typically 3–15 min per bed position), motion during the acquisition may lead to blurring in the resulting images and errors in quantification (1,2). The already limited spatial resolution of PET, around 4.5 mm in full width at half maximum, is effectively reduced when motion occurs during the acquisition. In oncology, tumors in the upper abdomen and thorax are particularly adversely affected by respiratory motion because the diaphragm moves by around 20 mm on average in one breathing cycle (3). Lesions at anatomic boundaries such as between the liver and lung can also be mispositioned on PET images when compared with the anatomic reference MR or CT image. Furthermore, quantification is affected because moving lesions appear smeared, showing an apparent increase in size and decrease in uptake. Motion may also cause problems with attenuation correction, whereby a static attenuation map does not correlate spatially with the PET emission data because of moving anatomy (4).

PET respiratory motion can be corrected by gating (splitting data into respiratory states), reconstructing separate images, and registering to a common respiratory state (2,5,6). This technique requires a good signal-to-noise ratio in each gated image for accurate registration. Meeting this requirement becomes difficult under the pressure to reduce scan times and lower patient doses, leading to low-count statistics and a lower signal-to-noise ratio in each gate. The recent advent of PET/MRI scanners allows exploitation of modality simultaneity by using high-contrast, high-spatial-resolution tagged MR images to estimate respiratory motion and correct PET data without additional radiation exposure, with MR tagging (7,8) or by acquiring quick motion-capturing 2-dimensional images (9) or low-resolution 3-dimensional images (10).

Although current methods for respiratory motion correction in PET/MRI show an improvement in PET image quality, all require a change to the otherwise intended PET/MRI protocol to be able to collect the respiratory signal or MRI-derived motion model in a clinical setting. Many methods use an external monitoring device to obtain a respiratory signal. However, such devices require time for set-up and readjustment and can fail because of mispositioning, patient movement, poor calibration, or signal drift and clipping. Some methods also require MRI-sequence alteration, which needs to be set up in advance of the scan, can create artifacts in MR images near the diaphragm, and may increase scan time.

In recent work, we demonstrated the capability of a joint PET/MRI-based predictive motion model that was built using data from a 1-min dynamic MRI sequence with no external hardware required (11). The method addressed many of the limitations found for the discrete binning method, which was used in our previous work (12). In this current work, we performed a pilot analysis of the method on a larger patient cohort by examining changes in SUV metrics on attenuation-corrected PET reconstructions and by performing a lesion detectability study. In this study, 2 readers examined each uncorrected and motion-corrected image and scored their confidence about suspected lesions. We used these scores to determine the true-positive (TP) and false-positive (FP) detection rates, as well as changes in confidence between uncorrected and motion-corrected images. Finally, we present several examples of how respiratory motion correction may have the potential to affect clinical patient management in such areas as staging, diagnosis, and surgical planning.

## MATERIALS AND METHODS

### Respiratory Motion Correction via Joint PET/MRI-Based Predictive Motion Model

In our recent work, we described a method of respiratory motion correction via a joint PET/MRI-based predictive motion model using 1 min of simultaneously acquired PET and MRI data to capture intercycle and intracycle breathing variations (11). The continuous nature of the model allows interpolation and extrapolation at any respiratory signal value, meaning 100% of PET data is used in the reconstruction, and deformation fields are estimated even at extreme values such as at a deep inhale for the Dixon MRI sequence. All slices of the free-breathing MRI acquisition are used, with an optimization scheme to form a model that is robust to registration errors between single slices. MRI and PET data were also aligned temporally by including a time shift in the optimization while taking the different hardware clock-rates into account. The motion model links one or more surrogate measures of respiratory motion to the tissue deformation. In our previous work, we found the performance to be best when we used the PET-derived respiratory signal and its gradient in a 2-surrogate model. The model used a linear fit to relate the surrogates to the deformations. This scheme was applied in the current work, on a larger patient dataset, to estimate deformations throughout the PET scans.

### Study Design

The U.K. Health Research Agency approved this retrospective study, and the requirement to obtain informed consent was waived. All data were acquired using an integrated 3-T PET/MRI system (Biograph mMR; Siemens Healthcare).

Data were retrospectively analyzed from 42 patients who had undergone PET/MRI between February 2014 and November 2015, selected on the basis of clinical information suggesting possible avid regions in the thorax or abdomen. The tracers used were ^18^F-FDG (24 patients) and ^68^Ga-DOTATATE (18 patients). The patient cohort consisted of 18 women and 24 men, with a mean age of 61.9 y (range, 36–85 y).

The PET/MRI protocols included an additional breath-hold DIXON MRI sequence (for PET attenuation correction) and a 1-min free-breathing dynamic MRI sequence (2-dimensional multislice gradient-echo sequence, with sagittal slices acquired at 9 locations, covering the thorax and abdomen, including the lungs, liver, and pancreas). The dynamic MRI sequence had the following parameters: 10-mm slice thickness, 25-mm gap between slice centers, 5.1-ms repetition time, 2.5-ms echo time, 10° flip angle, 965-Hz pixel bandwidth, 192 × 144 matrix, 262 × 349 mm field of view, 1.8 × 1.8 mm in-plane resolution, 0.3-s acquisition time per image, and factor 3 integrated parallel acquisition technique (Siemens Healthcare). The data from this 1-min MRI sequence were used to build the patient-specific motion model. Four consecutive minutes of PET list-mode data were used for the PET reconstructions, with a mean interval of 1 h 39 min ± 33 min from radiotracer injection to PET acquisition. The 4 min of PET data used here included the 1-min dynamic MRI acquisition and the preceding 3 min.

### Data Processing

A motion-compensated reconstruction of 4 min from the PET acquisition was performed using deformation fields estimated by the 2-surrogate linear model, with PET data gated before reconstruction using the patient-specific scheme outlined in a previous publication (11). For motion-corrected reconstructions, the attenuation μ-map was warped to each gate with deformation fields estimated by the motion model, using the values of the surrogate signal during the Dixon MRI acquisition. For uncorrected reconstructions, the acquired static μ-map was used. An ordered-subset expectation maximization reconstruction algorithm was applied, with 21 subsets, 3 iterations, 4-mm gaussian postfiltering, and correction of random events and scatter events.

PET data processing (e.g., unlisting and reconstruction) was performed with STIR (Software for Tomographic Image Reconstruction) (13). All other analyses were performed with Matlab (MathWorks, Inc.), and MIRT (Medical Image Registration Toolbox) (14) was used in Matlab for registration.

### Analysis

#### Lesion Detectability Study

The effect of motion correction on lesion detectability and localization was assessed. Two accredited radiologists viewed the uncorrected and motion-corrected PET images for each patient dataset without seeing the structural MR images. The viewing was done individually by each reader, and blinded to whether each image was uncorrected or corrected. The images were read in 2 sets, with the uncorrected and motion-corrected images for each patient split between the 2 sets randomly, and with at least a 2-wk interval between readings of each set to minimize recall bias. Each reader was free to scroll through the slices and adjust the color scales. The readers were asked to mark foci of uptake that might represent a pathologic change and influence clinical decisions. We use the term *lesion* to describe any marked area. The readers gave each mark a score, χ, on a 4-point scale to indicate their confidence that a lesion was present at that location (χ = 1, questionable [<50% likely]; χ = 2, possible [50%–75% likely]; χ = 3, probable [75%–95% likely]; χ = 4 [definite; >95% likely]). The scores provided by readers 1 and 2 are referred to as χ_1_ and χ_2_, respectively. The perceived anatomic location of each lesion on the uncorrected and motion-corrected PET images was also documented by the readers.

For the purpose of this study, the reference standard used to define the presence and locations of lesions was a consensus reading by the radiologists using a combination of all imaging studies, including the original uncorrected PET study, the MRI component of the hybrid PET/MRI study, contemporaneous clinical MRI and CT studies, and all available follow-up imaging (CT, MRI, PET). Lesions marked by the readers on the uncorrected and motion-corrected images were visually checked against the reference reading. Any marked lesion that matched a reference lesion was defined as a TP, any reference lesion that was not marked was defined as a false-negative and given a score of 0, and any marked lesion that did not match a reference lesion was defined as an FP.

A change in the χ score, Δχ, for any lesion in the TP or FP set was defined as the χ score for the motion-corrected image minus the χ score for the uncorrected image. For readers 1 and 2, these are referred to as Δχ_1_ and Δχ_2_, respectively. An increase in these scores after motion correction represents an increase in detection confidence, and a decrease represents a decrease in detection confidence.

#### SUV Analysis

Changes in 2 SUV metrics for lesions were assessed: ΔSUV_peak_, defined as the maximum average activity concentration within a 12-mm-diameter sphere inside a manually defined region of interest (15), and ΔSUV_max_, defined as the maximum voxel SUV inside the region of interest.

#### Statistical Analysis

The significance of differences in χ scores between uncorrected and motion-corrected images was determined with the Wilcoxon signed rank test. For the SUV metrics of lesions, a paired-sample *t* test was used.

## RESULTS

The reference reading identified 162 PET-positive lesions (74 for ^68^Ga-DOTATATE and 88 for ^18^F-FDG) in 32 patients. These were in the form of liver, pancreas, kidney, bowel, rib, and shoulder lesions, as well as an assortment of nodes and areas of benign uptake. These are summarized in Table 1. Ten patients had no identifiable lesions.

**TABLE 1:** Lesions in Patient Cohort

Lesion	*n*
Liver	71
Pancreas	22
Lung	27
Abdominal node	6
Thoracic node	29
Other*	7
Total	162

* Kidney, bowel, rib, or shoulder.

Of the 162 reference lesions, 72 were also confirmed to be present on MRI, 7 on CT, and 62 on both MRI and CT. Twenty-one lesions were confirmed either from PET follow-up or from the original uncorrected PET study along with patient history and clinical information. The quantitative analysis results are provided in Table 2.

**TABLE 2:** Quantitative Analysis Results for All 162 Lesions

Metric	Uncorrected	Motion-corrected	Paired significance
SUV_peak_	17.6 ± 18.0	19.5 ± 20.1	*P* < 0.0001 (*t* test)
SUV_max_	22.7 ± 22.6	26.6 ± 29.9	*P* < 0.002 (*t* test)
χ score	2.67 ± 1.50	3.01 ± 1.29	*P* < 0.0001 (Wilcoxon)
TP rate (%)	74	84	NA
FP lesions (*n*)	30	21	NA

NA = not applicable.

SUV metrics are means across all reference lesions. Detection score χ and TP rate are means across both readers. FP number is from 84 datasets (42 patients × 2 readers). Statistical significance is based on paired scores from uncorrected and motion-corrected datasets.

### Lesion Detectability

The TP rate, or sensitivity, for uncorrected and motion-corrected images was 85% and 95%, respectively, for reader 1 and 62% and 73%, respectively, for reader 2. The 2 readers’ average TP rate for the 162 lesions was 74% (^68^Ga-DOTATATE, 79%; ^18^F-FDG, 69%) in the uncorrected images, rising to 84% (^68^Ga-DOTATATE, 89%; ^18^F-FDG, 80%) in the motion-corrected images.

**FIGURE 1.**
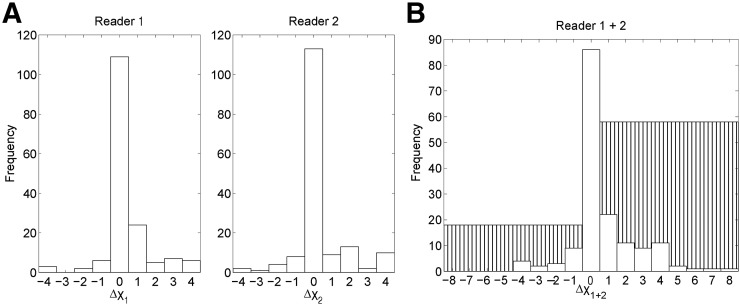
Change in detection score χ for TP lesions between uncorrected and motion-corrected images for readers 1 and 2 separately (A) and together (B). White bars represent number of lesions with specific score changes. Hatched bars are total number of lesions with negative or positive changes in score, where a positive change is “good” (i.e., TP lesions are more detectable after motion correction).

Figure 1 shows the Δχ scores for TP results, with a positive change indicating an increase in lesion detectability. Figure 1A shows the Δχ distribution for all 162 lesions for each reader separately. Considering the average of Δχ_1_ and Δχ_2_ over all 162 lesions, 8% (13 lesions) showed a decrease in χ score, 69% (112 lesions) showed no change, and 23% (37 lesions) showed an increase. Between the 2 readers, there was a significant increase in the mean χ score for TP results, from 2.67 (^68^Ga-DOTATATE, 2.92; ^18^F-FDG, 2.46) in the uncorrected images to 3.01 (^68^Ga-DOTATATE, 3.21; ^18^F-FDG, 2.85) in the motion-corrected images (*P* < 0.0001).

Figure 1B shows the summed χ scores, Δχ_1+2_, for each lesion. Overall, 11% (18 lesions) showed a decrease (range, −4 to −1), 53% (86 lesions) showed no change, and 36% (58 lesions) showed an increase (range, +1 to +8).

There was a significant increase in Δχ_1+2_ from the uncorrected images to the motion-corrected images, for all TP lesions (*P* < 0.0001).

We aimed to reduce the intrinsic intra- and interobserver variability of the scoring test by examining cases in which the Δχ_1_ and Δχ_2_ of a lesion were either both positive or both negative and in which the readers agreed on whether detectability had increased or decreased. This was the case for 14 lesions, with 1 lesion showing a negative change for both readers (range, −1 to −2) and 13 lesions showing a positive change for both readers (range, +1 to +4). For 4 of these lesions, the χ score changed from 0 in the uncorrected images (i.e., the lesions were invisible to both readers) to some degree of detectability in the motion-corrected images.

The total number of FP results, combining both readers, was 30 (^68^Ga-DOTATATE, 7; ^18^F-FDG, 23) in uncorrected images and 21 (^68^Ga-DOTATATE, 8; ^18^F-FDG, 13) in motion-corrected images. Overall, 27 lesions showed a decrease in χ score, 3 lesions showed no change, and 16 lesions showed an increase, for marked areas assumed not to be true lesions based on the reference reading.

### SUV Analysis

Over all 162 reference lesions, motion correction resulted in a significant increase in both SUV_peak_ (*P* < 0.0001) and SUV_max_ (*P* < 0.002), with a mean increase in SUV_peak_ of 12.4% (^68^Ga-DOTATATE, 12.6%; ^18^F-FDG, 12.2%) and a mean increase in SUV_max_ of 17.6% (^68^Ga-DOTATATE, 17.2%; ^18^F-FDG, 17.9%).

**FIGURE 2.**
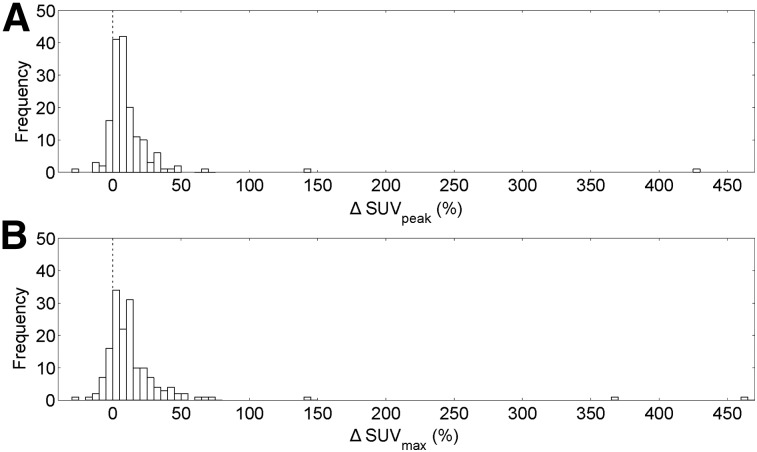
Histogram of all 162 reference lesions for the metrics ΔSUV_peak_ (A) and ΔSUV_max_ (B).

Figure 2 shows ΔSUV_peak_ and ΔSUV_max_ for all lesions. Of all lesions, 14% (22 lesions) showed a decrease in SUV_peak_ and 86% (140 lesions) showed an increase, whereas 17% (27 lesions) showed a decrease in SUV_max_ and 83% (135 lesions) showed an increase.

Defining a “considerable” change as a change greater than 5% for both ΔSUV_peak_ and ΔSUV_max_, 3% (5 lesions) showed a considerable decrease, 43% (69 lesions) showed an inconsiderable change, and 54% (88 lesions) showed a considerable increase. The 5 lesions that showed a considerable decrease were 1 lung node lesion, 1 lung lesion, 1 rib lesion, and 2 bowel lesions.

### Cross-Study Correlation

Overall, considering the 3 metrics ΔSUV_peak_, ΔSUV_max_, and Δχ_1+2_ for the 162 reference lesions, 2 lesions showed a decrease in all metrics and 49 showed an increase in all metrics. The lesions that showed a negative change were a mediastinal lymph node lesion and a bowel lesion.

### Clinical Case Studies

We present several case studies to show the effects of motion correction on PET images, considering potential to affect clinical management. The clinical MR images either were acquired at exhale breath-hold or were triggered, with data collected only at the exhale position. Uncorrected and motion-corrected images are displayed with the same color scale for each case study.

#### Case Study 1: New Lesions Detected, with MRI Confirmation

Case study 1 is a ^68^Ga-DOTATATE PET scan of a patient (falling within the age range of 70–80 y) who had recurrent neuroendocrine liver metastases after partial hepatectomy. A contemporaneous MRI study showed at least 2 suspected lesions in the remnant liver and several smaller deposits that were of concern.

**FIGURE 3.**
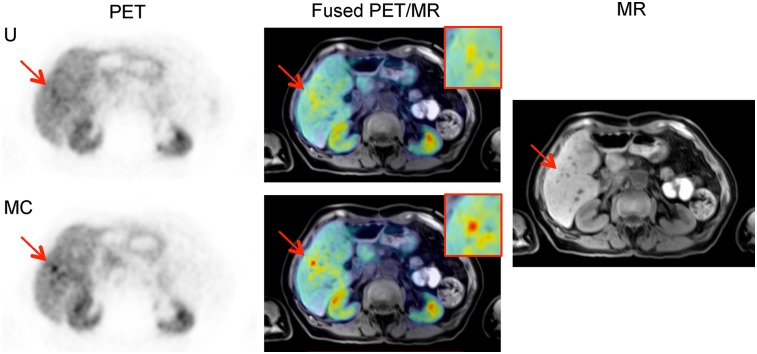
Case 1. Axial uncorrected (U) and motion-corrected (MC) PET images of lesion 1, along with T1-weighted Dixon volumetric interpolated breath-hold examination PET/MR image and MR image alone.

In total, the Δχ_1+2_ scores for 3 very small lesions was [+1,+1,+1], with 6 of the total readings (3 lesions × 2 readers) showing that the 3 lesions were newly detected in the motion-corrected images. All 3 were found to be present in the MR images in the reference reading. Figure 3A shows one of the newly detected lesions in uncorrected and motion-corrected images, with the contrast of the lesion being much higher in the motion-corrected image. The SUV results were confirmatory, with increases in the SUV metrics for the 3 lesions (Fig. 3B).

At least 3 lesions showed DOTATATE avidity, suggesting metastatic neuroendocrine lesions. This information would influence decisions on the choice of treatment, which may be drug treatment (e.g., octreotide analog), percutaneous ablation, or resection.

#### Case Study 2: New Lesions Detected, with PET Follow-up Confirmation

Case study 2 is a ^68^Ga-DOTATATE PET scan of a patient (falling within the age range of 40–50 y) known to have multiple endocrine neoplasia syndrome type 1 and pancreatic lesions. A PET/MRI scan was requested to assess lesion uptake and determine the possible surgical approaches.

**FIGURE 4.**
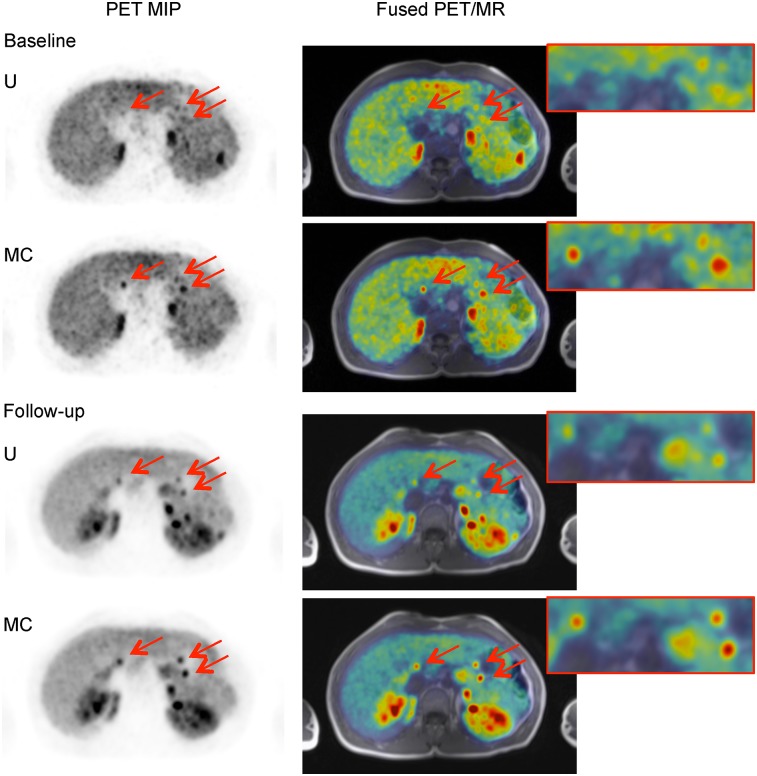
Case 2. Axial uncorrected (U) and motion-corrected (MC) PET maximum-intensity projection (MIP) images showing 3 pancreas lesions (arrows) at baseline and follow-up, along with T1-weighted Dixon volumetric interpolated breath-hold examination PET/MR images.

Although the lesions were not verified in the MR images available, 6 lesions were confirmed as present in the reference reading because of visibility in a follow-up scan performed 1 y later (Fig. 4).

The increased number of detected lesions in the baseline scan is crucial. Accurate mapping of the number and location of tumors in the pancreas is critical for evaluating the risk versus the benefit of surgical intervention and for planning the surgery if pursued. The larger the extent of the pancreatectomy, the more complex the surgery would be and the greater would be the risk of subsequent diabetes as a complication.

#### Case Study 3: Change in Lesion Location

Case study 3 is an ^18^F-FDG PET scan of a patient (falling within the age range of 40–50 y) with known liver metastases found in a previously acquired CT scan.

**FIGURE 5.**
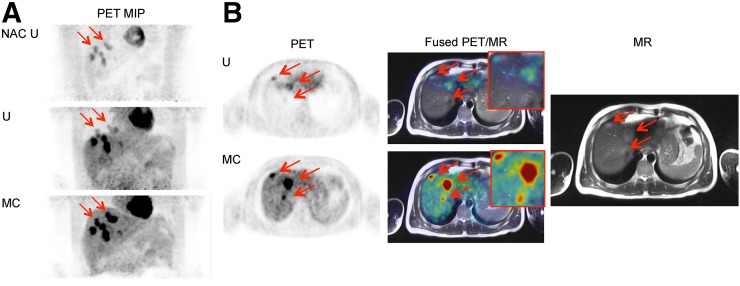
Case 3. (A) Maximum-intensity projection (MIP) non–attenuation-corrected, non–motion-corrected (NAC U); attenuation-corrected, non–motion-corrected (U); and attenuation-corrected, motion-corrected (MC) PET images. (B) Axial PET slice with 3 lesions (arrows) that wrongly appear in lung in uncorrected image (U) and correctly appear in liver in motion-corrected image (MC), along with T2-weighted half-Fourier–acquired single-shot turbo spin-echo PET/MR images and MR image alone.

For one reader, the location of the lesion changed from lung to liver after motion correction. The reference reading for this patient showed 8 lesions, all of which were confirmed to be in the liver on the MR images. In PET maximum-intensity projections (Fig. 5A), the location of 2 lesions at the lung–liver interface was unclear in both the non–attenuation-corrected and the attenuation-corrected images, but the motion-corrected image clearly showed that the lesions were in the liver. The spatial alignment of the fused PET and MR images was better when motion was corrected than when it was not (Fig. 5B).

This large change in location was due to inadvertent acquisition of the Dixon MR image at a deep exhale. Motion correction of the resulting misalignment meant a large ΔSUV_peak_ (+142% and +366%) for the lesions that appeared to move from the lung in the uncorrected image to the liver in the motion-corrected image.

Motion correction of lesion localization is important for staging and treatment planning. Involvement of more organs by metastases could potentially change disease stage, influence treatment decisions, and imply a different prognosis.

#### Case Study 4: Change in Intralesional Activity Distribution

**FIGURE 6.**
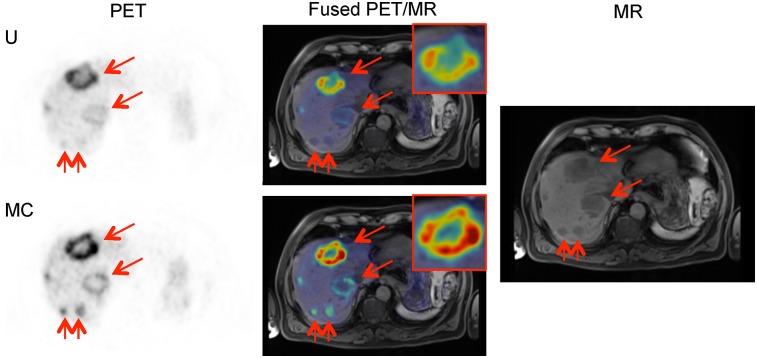
Case 4. Coronal and axial uncorrected (U) and motion-corrected (MC) PET images, along with T1-weighted volumetric interpolated breath-hold examination spectral attenuated inversion recovery PET/MR images showing change in shape of uptake in necrotic lesion, and MR image alone. Arrows show the area where there was artifacts, which are no longer present in MC image.

Case study 4 is a ^68^Ga-DOTATATE PET scan of a patient (falling within the age range of 60–70 y) in whom the χ scores showed either no change or a slight increase in the 11 lesions identified in the reference reading. The shape of the activity distribution in 3 of these lesions was changed by motion correction (Fig. 6). These were necrotic lesions showing uptake only at the outer edge.

Although lesion detection and localization did not significantly change, the distribution of intralesional uptake may be of clinical importance. For example, a change in distribution may influence the perceived optimal site for PET-directed biopsy in some patients or for PET-guided intensity-modulated radiation therapy in others.

#### Case Study 5: Artifact Reduction

**FIGURE 7.**
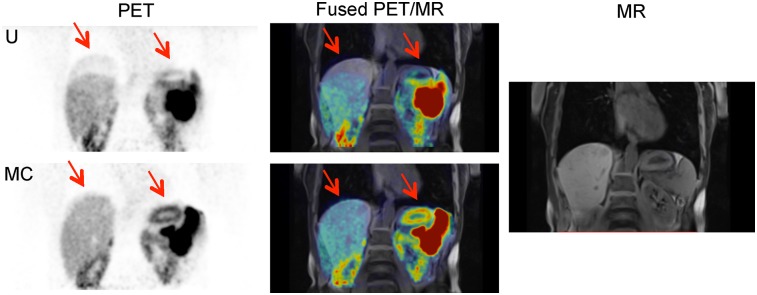
Case 5. Coronal uncorrected (U) and motion-corrected (MC) PET images, along with T1-weighted volumetric interpolated breath-hold examination PET/MR images showing reduction in attenuation misalignment artifacts (arrows), and MR image alone.

Case study 5 is a ^68^Ga-DOTATATE PET scan of a patient (falling within the age range of 50–60 y) whose uncorrected image showed a banana artifact at the top of the liver due to a misaligned attenuation map (Fig. 7). Removal of the artifact was clearly seen in the motion-corrected image, as well as restoration of the shape of the high stomach uptake to the shape seen in the MR image. The motion-corrected PET image spatially aligned better with the MR image.

## DISCUSSION

This work constitutes a pilot clinical validation of our joint PET/MRI-based predictive motion model. The PET respiratory motion correction method produced significant increases in all tested metrics in reference lesions, with mean increases of 12.4% and 17.6% in SUV_peak_ and SUV_max_, respectively, in 162 PET-avid lesions. We also showed an increase in the χ scores of readers in detecting avid lesions, with a mean score of 2.67 rising to 3.01 through motion correction, and a TP rate of 74% rising to 84%. Only 2 lesions showed a decrease in all 3 metrics (SUV_peak_, SUV_max_ and Δχ_1+2_), whereas 49 lesions showed an increase in all metrics.

Several clinical examples were presented to demonstrate the range of positive effects of respiratory motion correction with our method, including newly detected lesions, increased lesion sharpness, reduced artifacts, improved lesion localization, and improved visualization of intralesional activity distribution.

In the SUV analysis (Fig. 2), the apparent outliers arose from good results, with the large increases in lesion SUV_peak_ and SUV_max_ being due to correction of attenuation map misalignment. In the only 5 lesions that showed a considerable (more than 5%) decrease in SUV, the decrease could have been due to their location. Two of these lesions (including that with the largest decrease, 25%) were in the bowel, an area in which the model cannot predict motion because bowel motion is sporadic and unrelated to respiration. Another lesion was on a rib, a location in which the deformation estimation might be poor because the registration scheme lacks a sliding motion. The remaining 2 lesions were in the lung of a patient with a large lung mass, potentially causing unpredictable breathing patterns.

When the combined χ scores from both readers were considered, 11% of lesions showed a decrease in detectability and 36% showed an increase, with the range of χ scores being much smaller in the decreased-detectability set (−4 to −1, vs. +1 to +8).

One limitation of the detectability study was intra- and interobserver variability in interpretation. The interobserver variability was higher than might be expected for a PET study focusing on a specific clinical context. However, the study used multiple cohorts of patients and diseases. In addition, the detectability assessment was initially done in isolation, without clinical patient information or structural information from MRI or CT. We attempted to overcome this limitation by analyzing the results from both readers together. For example, when we looked at only the results for which Δχ was either positive or negative for both readers in all reference lesions, we found that 13 lesions showed an increase in detectability and only 1 showed a decrease. However, this single lesion had a higher SUV_peak_ and SUV_max_ in the motion-corrected image than in the uncorrected image, suggesting that the negative Δχ was also due to human error.

A recommended approach for testing lesion detectability is free-response receiver-operating-characteristic analysis (16). We did not perform such an analysis because our study lacked a consistent reference standard and had no definite method to identify FPs. In the literature, PET-based detection studies revolving around testing of different reconstruction methods (17), different acquisition times (18), different methods of motion correction (19), or time-of-flight impact (20) use phantom or simulated PET data for which ground truth is known. The information that PET provides is unique in that it portrays tracer uptake, which is unique to the modality. For example, a lesion may appear avid in a PET image but may not necessarily be visible in an MR or CT image.

The lack of a definite way to define FPs (a PET-avid lesion that is not apparent on MRI or CT) applies to our result for the FP rate (30 lesions detected in the uncorrected images and 21 in the motion-corrected images, from 84 datasets [42 patients × 2 readers]). These lesions were marked as FPs because evidence of a true lesion was lacking in the patient information or in the results of other imaging modalities, but in reality, some of these FPs may have been real lesions.

We consider this study a pilot analysis of our method. With a streamlined pipeline and enhanced data-processing efficiency, the method can be adopted into routine practice, which in turn would provide the substrate needed for wider clinical validation.

## CONCLUSION

We have demonstrated significant improvements in quantification and detection of PET-avid lesions using multiple tracers and in multiple organ locations, with specific case examples showing where motion correction has the potential to affect diagnosis or patient care.

## DISCLOSURE

Funding was provided by a Siemens/UCL IMPACT studentship, EPSRC (EP/K005278/1), and the NIHR University College London Biomedical Research Centre of the National Institute for Health Research. No other potential conflict of interest relevant to this article was reported.
